# Microwave-Assisted Extraction of *Curcuma longa* L. Oil: Optimization, Chemical Structure and Composition, Antioxidant Activity and Comparison with Conventional Soxhlet Extraction

**DOI:** 10.3390/molecules26061516

**Published:** 2021-03-10

**Authors:** Rut Fernández-Marín, Susana C. M. Fernandes, María A. Andrés, Jalel Labidi

**Affiliations:** 1 Environmental and Chemical Engineering Department, University of the Basque Country UPV/EHU, Plaza Europa 1, 20018 Donostia-San Sebastián, Spain; rut.fernandez@ehu.eus (R.F.-M.); marian.andres@ehu.eus (M.A.A.); 2 IPREM, E2S UPPA, Universite de Pau et des Pays de l’Adour, CNRS, 64600 Anglet, France; susana.fernandes@univ-pau.fr

**Keywords:** *Curcuma longa* L., antioxidant properties, phenolic content, microwave-assisted extraction, surface response methodology, experimental Box-Behnken design

## Abstract

Curcuma root (*Curcuma longa* L.) is a very important plant in gastronomy and medicine for its unique antiseptic, anti-inflammatory, antimicrobial and antioxidant properties. Conventional methods for the extraction of curcuma oil require long extraction times and high temperatures that can degrade the active substances. Therefore, the objectives of the present study were: (i) first, to optimize the extraction yield of curcuma oil by applying a Box-Behnken experimental design using surface response methodology to the microwave-assisted extraction (MAE) technique (the independent variables studied were reaction time (10–30 min), microwave power (150–200 W) and curcuma powder/ethanol ratio (1:5–1:20; *w*/*v*); and, (ii) second, to assess the total phenolic content (TPC) and their antioxidant activity of the oil (at the optimum conditions point) and compare with the conventional Soxhlet technique. The optimum conditions for the MAE were found to be 29.99 min, 160 W and 1:20 *w*/*v* to obtain an optimum yield of 10.32%. Interestingly, the oil extracted by microwave-assisted extraction showed higher TPC and better antioxidant properties than the oil extracted with conventional Soxhlet technique. Thus, it was demonstrated that the method applied for extraction influences the final properties of the extracted *Curcuma longa* L. oil.

## 1. Introduction

Curcuma or turmeric root (*Curcuma longa* L.) is an herbaceous plant that belongs to the *Zingiberaceae* family. Its cultivation is widespread in tropical and subtropical regions, especially in Asia, being India and China the greatest producers [1,2,3]. Turmeric is known since antiquity and is of great importance worldwide in different applications namely in gastronomy as a condiment, in the textile industry as a dye and in medicine as anti-inflammatory [4,5] or anti-cancer treatment [6], or in the treatment of Alzheimer’s disease [7,8]. It is also used as antiseptic [2], antioxidant [9], antiviral [10], antimicrobial [4,5], as well as an insect repellent [11,12].

This is mainly due to the presence of the oleoresins of turmeric, which are a mixture of curcuminoids and essential oils. Curcuminoids are yellow pigments whose main compounds are curcumin (70–75%), demethoxycurcumin (10–20%) and bisdemethoxycurcumin (5–10%) and represent 2–9% of the active components in turmeric [2,13,14]. Essential oils, that represent 3–5% of active compounds, are aromatic, volatile liquids with ar-turmerone as the major compound, which is a class of sesquiterpenoid cyclic ketone [3,13,15].

Due to their great importance in several applications, different extraction approaches have been developed, usually. These compounds are usually extracted by steam distillation, hydrotrope, hot and cold percoxation, Soxhlet and alkaline solution, which are known as conventional methods [2,16,17]. Nevertheless, these methods require high energy, temperatures and solvent consumption, in addition to long reaction times leading consequently to the degradation of many compounds and low yields [2,18]. Therefore, eco-friendly alternatives including ultrasound assisted extraction, supercritical fluids (mainly supercritical carbon dioxide) and microwave assisted extraction (MAE) have been studied [17,19,20,21].

Among them, the MAE technique has proven to be a good alternative for the extraction of bioactive compounds from plants due to the low energy and solvent consumption, short reaction times and high extraction yields. In 2011, Wakte and co-authors compered the efficiency of different extraction techniques, including MAE, Soxhlet, ultra-sonic and supercritical CO_2_ assisted extraction in terms of yield. In this work, they demonstrated that MAE technique was more efficient for the curcumin extraction from powdered *C. longa* rhizomes [17].

Herein, we go a step further: first, by optimizing the MAE conditions using response surface methodology (RSM). RSM has shown to be a useful multivariate technique for the optimization of complex experimental processes with many factors. The RSM is a mixture of statistical and mathematical modeling techniques, evaluating the effect of several independent variables with a view to determine the optimal value for the desired product. This methodology is very practical since the regression equations of the variables under study are obtained, as well as the responses in the desired ranges. Moreover, the relationships between the dependent and independent variables can be observed by the use of contour plots [22]. In addition, a Box-Behnken design is employed to investigate the effects of the microwave power, *Curcuma longa* L./EtOH ratio, and extraction time; and second, by comparing the yield, total phenolic content and the antioxidant properties of *Curcuma longa* L. oil extracted by MAE and Soxhlet.

## 2. Results and Discussion

### 2.1. MAE Based Experimental Design

#### 2.1.1. Optimization of the *Curcuma longa* L. Oil Extraction

Conventional methods to extract *Curcuma longa* L. oil require long reaction time and high-energy consumption. These conditions often cause degradation of the extracted compounds and low yields. Therefore, it is important to use other extraction techniques, in particular MAE and optimize the extraction conditions to obtain better yields while maintaining the quality of the extracted samples. For this purpose, a BBD experimental design with the response surface method was used to optimise the extraction yield of *Curcuma longa* L. oil. In the present study, the independent variables were the reaction time (t, min), the microwave power (P, W) and the ratio *w*/*v* (R, g/mL). The dependent variable was the yield of the extracted *Curcuma longa L.* oil (Materials and Methods section). Table 1 lists the experiments randomly determined by Statgraphics Centurion software (StatPoint Technologies Inc., Warrento, VA, USA) and the experimental results of the extraction yield (dependent variable).

The validation of the model was determined by the regression coefficient (*R^2^*) explaining the total variations of the model and was reinforced by Fisher’s F-test obtained by ANOVA [23]. As showed in Table 2, the value of R^2^ was 0.82, which means that only 0.18% of the total variances are not explained by the selected model. Regarding Fisher’s F-test, the high F values confirm a good fit of the model considering that the experimental F (2.55) was higher than the critical F (0.16) with nine degrees of freedom. This indicates that the values obtained for the proposed model are appropriate to describe the interactions between the different variables selected.

Concerning the regression coefficients, the variables microwave power, *Curcuma longa* L. powder/EtOH ratio and the quadratic of power significantly influenced the oil extraction yield (Table 2). Power and *Curcuma longa* L. powder/EtOH ratio showed an influence with a confidence interval of 95%, while in the case of quadratic power it was 90%. Through the obtained regression coefficients, the second-degree polynomial equation for the extraction yield has been calculated as shown below:

Y_C-MAE_ % = 9.44 + 0.32X_1_ − 1.40X_2_ – 1.39X_3_ – 0.99X_1_^2^ – 0.55X_1_X_2_ – 1.23X_1_X_3_ – 1.65X_2_^2^ + 0.66X_2_X_3_ – 1.51X_3_^2^ (1)

#### 2.1.2. Effect of Independent Variables on Extraction Yield Response

**Figure 1:**
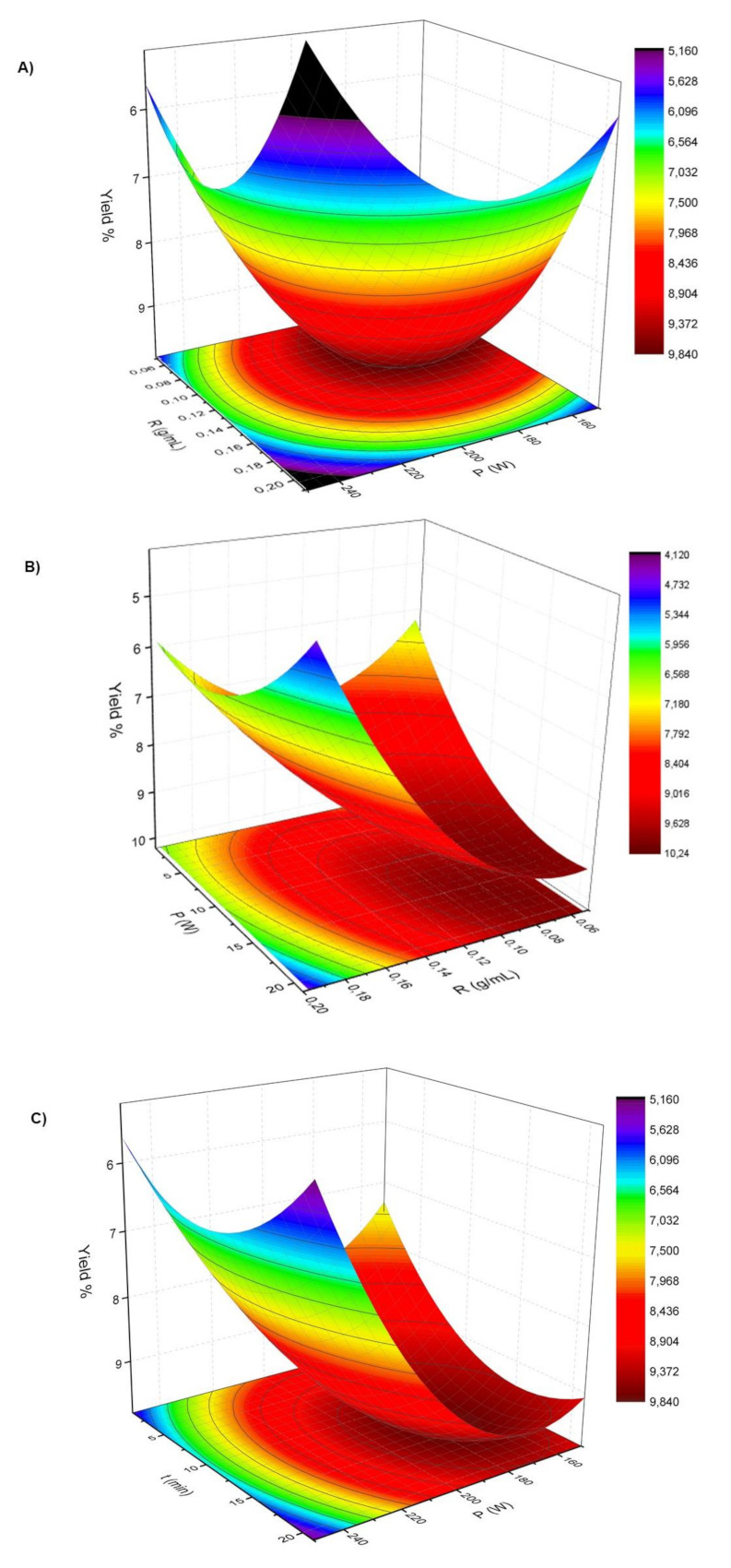
*Curcuma longa L.* oil extraction yield as a function of: (**A**) P (power, W) and R (*Curcuma longa* L. powder/EtOH ratio, g/mL) at a fixed t (time, min, X_1_ = 0); (**B**) t (time, min) and R (*Curcuma longa* L. powder/EtOH ratio, g/mL) at a fixed P (power, W, X_2_ = 0); (**C**) t (time, min) and P (power, W) at a fixed R (*Curcuma longa* L. powder/EtOH ratio, g/mL, X_3_ = 0).

As shown in Table 1 the extraction yields of *Curcuma longa* L. oils were found to be in the range of 3.03% (exp.12) and 10.84% (exp.11). The surface plot (Figure 1A–C) presents the interactions between the independent variables (t, P and R) and the extraction yield of *Curcuma longa* L. oil by MAE (Y_C-MAE_).

Figure 1A shows the influence of the independent variables microwave power (X_2_) and *Curcuma longa* L. powder/EtOH ratio (X_3_) on the extraction yield for a fixed extraction time at a mean value (X_1_ = 0). This surface plot shows how the extraction yield increases when P (X_2_) and R (X_3_) decrease. In this design, the extraction yield reaches a maximum when microwave power (X_2_) is around 155 W and *Curcuma longa* L. powder/EtOH ratio approximately 1:20. However, these parameters rise above these values when yield decreases which reaffirms the negative quadratic effect of P^2^ (Table 2). As listed in Table 2, these two independent variables have an influence on the model.

Figure 1B displays the extraction yield as a function of time (X_1_) and *Curcuma longa* L. powder/EtOH ratio (X_3_) keeping the microwave power constant at a midpoint value (X_2_ = 0). The progressive decrease of *Curcuma longa* L. powder/EtOH ratio and the increase of extraction time resulted in a positive effect on the extraction yield of *Curcuma longa L.* oil. The extraction yield obtained a maximum value at around 10%, when high reaction time and a minimum amount of *Curcuma longa L.* powder were used.

Figure 1C presents the response surface for extraction yield as a dependence of reaction time (X_1_) and microwave power (X_2_) for a fixed value of *Curcuma longa* L. powder/EtOH ratio (X_3_ = 0). This curve discloses a highest extraction yield when the extraction time increases and the microwave power is decreases. This trend is explained by the linear and quadratic negative contribution of power (Table 2).

#### 2.1.3. Comparison of MAE and Soxhlet Extraction Yields

The objective of the BBD experimental design was to optimize the different extraction conditions i.e., extraction time, microwave power and *Curcuma longa* L. powder to obtain the maximum extraction yield of *Curcuma longa* L. oil. The expected optimum point values were calculated using Statgraphics Centurion XV software and experimentally verified in triplicate in order to validate the model (Table 3).

As listed in Table 3, the optimum extraction yield obtained experimentally was 10.32 ± 0.69%, while the predicted extraction yield calculated by the software was of 10.92%. The proximity of these two values confirms the validation of this BBD design. This optimum extraction yield of 10.32 ± 0.69% was attained using 1:20 g/mL ratio of *Curcuma longa* L./EtOH for 30 min and with a microwave power of 160 W.

From the Soxhlet extraction technique, a yield value of 8.44 ± 0.17% was obtained (Table 3) using 5 g of *Curcuma longa* L. in 150 mL ethanol for 6 h. Vijayan et al., obtained similar *Curcuma aromatic* extraction yield (7.48%) by using the same extraction technique and solvent [24]. Priyanka et al., observed lower extraction yields of *Curcuma longa* (5.95%) using a non-polar solvent like *n*-hexane for 24 h [25].

Comparing the MAE with the conventional Sohxlet technique (Table 3), the first technique showed better extraction yield of *Curcuma longa* L. oil together with a reduction of the extraction time (from 6 h with Sohxlet method to 30 min with MAE) and lower energy consumption. Wakte and co-authors, compared different techniques such as ultra-sound, Soxhlet, supercritical CO_2_ and MAE, they have observed high extraction yields of curcuminoid compounds with shorter times using the MAE technique [17].

### 2.2. Characterization of the Extracted Curcuma longa L. Oil

In the next sections, the chemical structure and composition of the *Curcuma longa* L. oil obtained by the two techniques are assessed and compared as well as the amount of total phenolic content and antioxidant properties.

#### 2.2.1. Chemical Structure and Composition

**Figure 2:**
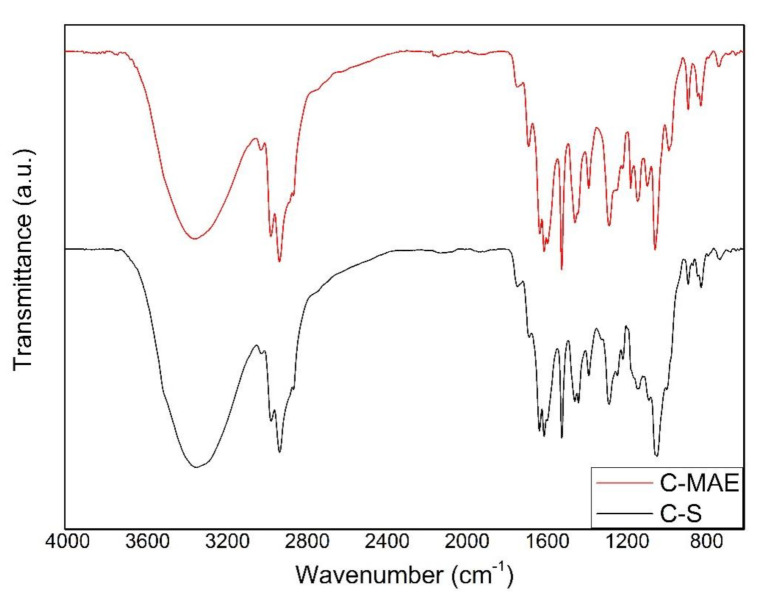
ATR-FTIR spectra of the *Curcuma longa* L. oil extracted with microwave-assisted extraction (C-MAE) and Soxhlet method (C-S).

Figure 2 displays the ATR-FTIR spectra of *Curcuma longa* L. oils obtained by the MAE and Soxhlet methods. The two samples exhibited identical spectra, showing that similar compounds were obtained by both methods. The two samples showed a broad band around 3340 cm^−1^ corresponding to O–H stretching. The -CH_3_ and -CH_2_ stretching vibrations were observed around 2967 and 2925 cm^−1^, respectively [26,27]. The peak around 1680 cm^−1^ was attributed to C=O vibrations [27] and the peaks at 1624 cm^−1^ and 1600 cm^−1^ were assigned to the aromatic ring stretching (C=C) and to a mixture of C=C and C=O stretching, respectively [20,26,27]. The band at around 1430 cm^−1^ was ascribed to CH_2_ bending, while the peak around 1377 cm^−1^ was assigned to CH_3_ bending indicating the presence of curcuminoids [20]. The band observed near to 1034 cm^−1^ was attributed to C-OH stretching vibration [26]. The small peaks appearing around 879 cm^−1^ and 814 cm^−1^ were assigned to C–O vibrations and C–H vibrations out of the plane of the aromatic ring. These two peaks are important considering that they are characteristic of the species *Curcuma longa* L. [26,27].

Table 4 summarizes the volatile components of *Curcuma longa* L. oil extracted by the MAE and Soxhlet methods. The identification of the compounds was done by using the NIST library and the stat-of-the-art in this domain. Interestingly, the same compounds were obtained in almost the same proportions for both techniques. The main components obtained were ar-turmerone (33.78% MAE and 37.08% Soxhlet), turmerone (20.12% MAE and 18.15% Soxhlet) and β-turmerone, known as curlone (20.05% MAE and 19.22% Soxhlet). These data were comparable to those reported before in the literature using different extraction techniques such as hydro-distillation, different supercritical fluids and especially the use of CO_2_ with co-solvents [3,18,20,28].

#### 2.2.2. Total Phenolic Content and Antioxidant Activity

Table 5 shows the TPC and antioxidant activity data of the *Curcuma longa* L. oils obtained at the optimum point of the experimental design with the MAE and with the conventional Soxhlet extraction.

Interestingly, these data show that at the optimal point with MAE technique, higher phenolic compound content were obtained when compared with the Soxhlet method: 232.75 ± 0.31 and 140.72 ± 0.42 mg GAE/g *Curcuma longa* L. extracted oil for MAE and Soxhlet, respectively. These results were higher than those obtained by de Carvalho and co-authors (5018 mg GAE/100 g), that extracted *Curcuma longa* L. oil by supercritical fluid method with CO_2_ and EtOH as co-solvent [29]. Patil et al., obtained similar values for turmeric extraction by using the Soxhlet method (143.67 mg GAE/g) [30].

The lower TPC with Soxhlet may be due to: (i) the long exposure (6 h) of the sample to high temperatures (solvent boiling temperature) and consequently degradation of the sample [31]; and (ii) the different of MAE and Soxhlet process; i.e., the electromagnetic field generated in the MAE method induced high pressures leading to the cell walls disruption allowing a better diffusion of the substances [32,33]. Similar trends were reported by Sánchez-Reinoso and co-authors, who used the MAE and Soxhlet extraction method for the extraction of *Sacha inchi* shell extracts [32]. Also, Pan et al., observed the same fact when extracting oil from *Osmanthus fragans* flower using MAE with deep eutectic solvents and reflux extraction with ethanol [34].

The antioxidant activity results from a synergy among the different phenolic compounds. Because the *Curcuma longa* L. antioxidant activity results from different mechanisms, in the present study, three methods were employed (DPPH, FRAP and ABTS) to compare the two extraction methods.

Among the three techniques, DPPH showed the lowest antioxidant activity. Similar results were observed previously, in the extraction of *Curcuma longa* oil by Soxhlet vs. supercritical fluid CO_2_ and ethanol [29]. As previously observed in oil extracted from seeds and flowers [29,32], with the FRAP assay a considerable difference was detected in the antioxidant activity between the samples extracted by MAE (255.66 ± 0.24 mg GAE/g) and Soxhlet (73.37 ± 0.18 mg GAE/g) (Table 5). Finally, the ABTS method is considered excellent for the evaluation of the antioxidant activity of several substances and can be applied to both liposoluble and hydrosoluble compounds. The results obtained for the ABTS test with MAE and Soxhlet, even if quite similar, showed a significant difference *p* < 0.05 between both techniques. Other authors showed lower values of ABTS in the extraction of *Curcuma longa* using supercritical fluid CO_2_ and ethanol [29] compared with our data.

The differences in the values between the antioxidant assays could be due to the different mechanisms involved. The DPPH assay measures the ability of a substance to donate a hydrogen to the DPPH* free radical, whereas the FRAP method is based on the measurement of the reduction of the ferric ion-TPTZ complex. In the ABTS assay, the activity is based on the capacity of the *Curcuma longa* L. oil to decrease the amount of ABTS^•+^ cation radical preformed in the solution. This reducing capacity of *Curcuma longa* L. oil is an important indicator of their antioxidant capacity [34].

Taking into account the obtained data, it can be concluded that the MAE technique provides samples with higher TPC and this phenolic extract exhibited higher antioxidant properties compared with traditional methods.

## 3. Materials and Methods

### 3.1. Raw Materials and Chemicals

Curcuma root (*Curcuma longa* L.) from India was purchased in a local market in Bizkaia, Spain. The root was washed in water to remove impurities and dried at 50 ± 0.05 °C. Then, the root was milled with a cutting mill (Retsch SM 2000, Haan, Germany) and was sieved with a 0.5 × 0.5 mm mesh. The powder obtained from the curcuma root was stored in the dark at room temperature.

Ethanol (EtOH, analytical standard), ethyl acetate (C_4_H_8_O_2_, HPLC grade), methanol (MeOH, HPLC grade), 2,2-diphenyl-1-picrylhydrazyl (DPPH) and 6-hydroxy-2,5,7,8-tetramethylchromane-2-carboxylic acid (Trolox), bis(3-ethylbenzothiazoline-6-sulphonic acid) (ABTS) and 2,4,6-tris(2-pyridyl)-s-triazine (TPTZ) were purchased from Sigma-Aldrich (Madrid, Spain). Glacial acetic acid (CHCOOH, technical grade), sodium chloride (NaCl), sodium acetate (CH_3_COONa), sodium hydrogen phosphate (Na_2_HPO_4_), potassium dihydrogen phosphate (KH_2_PO_4_), potassium chloride (KCl), potassium peroxodisulphate (K_2_S_2_O_8_) and hydrochloric acid (HCl, 37%) were purchased from Panreac AppliChem (Barcelona, Spain). Sodium carbonate anhydrous (NaCO, general-purpose grade) was supplied by Fisher (Madrid, Spain) and Iron (III) chloride hexahydrate (FeCl_3˙_6H_2_O) was obtained from Acros Organics (Madrid, Spain). Gallic acid monohydrate (C_7_H_6_O_5˙_H_2_O, extra pure) and Folin-Ciocalteu reagent was obtained from Scharlau (Barcelona, Spain).

### 3.2. Extraction of Curcuma longa L. Oil

#### 3.2.1. Microwave-Assisted Extraction (MAE)–Experimental Design

##### General Experiment Procedure

The MAE extraction was carried out in open vessel equipped with a condenser and heated by microwave (flexiWAVE, Milestone, Sorisole, Italy). The extractions were performed by using a *Curcuma longa L.*/ EtOH ratio *w*/*v* of (g/mL) employing a ratio range of 1:20–1:5 g/mL (Table 1 and Table 6). After the extractions, the solvent was removed with a rotary vacuum evaporator. The extraction yield was determined gravimetrically and the experiment at optimum point was triplicated.

##### Experimental Design and Determination of the Optimal Extraction Conditions

Response surface methodology (RSM) was applied to analyse the effect of the reaction time (t, min), the microwave power (P, W) and the *Curcuma longa* L./EtOH ratio *w*/*v* (R, g/mL) on the extraction yield (Y). For the experimental design and optimisation, a Box-Behnken Design (BBD) was used, consisting of 15 experiments of which three correspond to replicates of the central point. The experimental design was prepared and the optimal conditions were predicted with the desirability function of the Statgraphics Centurion version XV software (StatPoint Technologies INC., Warrento, VA, USA).

Table 6 shows the experimental variables (independents and dependents) used in the design and their values or range.

The regression analysis function of Microsoft Excel Add-In (Microsoft, Washington, DC, WA, USA) was implemented to adjust the experimental data obtained in the experimental design. To adjust the experimental data, a second order polynomial equation was employed:

(2) $$y_{j}=\beta_{0}+\overset{k}{\underset{i\;=\;1}{\sum }}\beta_{i}X_{i}+\overset{k}{\underset{i=1}{\sum }}\beta_{ii}X_{i}^{2}+\sum \overset{k}{\underset{i<j\;=\;1}{\sum }}\beta_{ij}X_{i}X_{j}+\varepsilon$$

where Y is the dependent variable *Curcuma longa L.* oil yield (%), K corresponds to the number of factors (3), ε is the experimental error, β_0_, β_i_, β_ii_ and β_ij_ indicate the regression coefficient estimated from the experimental results by the method of minimum squares, and Xi and Xj are the dimensionless ones and indicate the standardised independent variables (variation range from −1 to 1).

The model was validated by evaluating the lack of fit, the coefficient of determination (*R^2^*), the importance of the regression coefficients, the value of the F test using analysis of variance (ANOVA).

#### 3.2.2. Soxhlet Extraction

The Soxhlet (conventional method) extraction was done following the method [20] with slight modifications. For the extraction, 5 g of *Curcuma longa* L. powder were put in 150 mL of EtOH at boiling temperature (78 °C) for 6 h. After extraction, the solvent was then removed using rotary vacuum evaporator at 50 °C until a constant weight in order to obtain the extraction yield gravimetrically. The experiments were done in triplicate.

### 3.3. Characterization of the Curcuma longa L. Oil

The *Curcuma longa* L. oil samples obtained by MAE in the optimum point, and by Soxhlet extraction were characterized in terms of chemical structure and composition as well as their total phenolic content and antioxidant properties.

#### 3.3.1. Attenuated Total Reflection-Fourier Transform Infrared Radiation (ATR-FTIR)

Attenuated Total Reflection-Fourier Transform Infrared Radiation (ATR-FTIR) was employed to determine the chemical structure of the *Curcuma longa* L. oil. For this purpose, a spectrum-two FTIR spectrometer (Perkin Elmer Inc., Waltham, MA, USA) with a Universal Attenuated Total Reflection accessory was used. The transmittance range was from 600 to 4000 cm^−1^ using 32 scans and 4 cm^−1^ of resolution.

#### 3.3.2. GC/MS Analysis

The chemical composition of the extracted *Curcuma longa* L. oil was assessed using gas chromatography and mass spectrometry (GC/MS, GC 7890A, MS 5975C, Agilent, Santa Clara, CA, USA). First, 0.01 g of *Curcuma longa* L. oil was dissolved in 1 mL of ethyl acetate, and, then this solution was injected into a HP-5MS capillary column (5%-phenyl)-methylpolysiloxane, 30 m × 0.25 mm). The conditions used to carry out the GC/MS analysis were as following described: the injector was at 280 °C with split/splitless method in split mode (10:1); the carrier gas was helium with a flow rate of 0.7 mL/min; the temperature ramp started at 50 °C and was increased by 10 °C/min until reaching 120 °C and maintained for 5 min; in the second stage of the ramp, the temperature was increased by 10 °C/min until 280 °C and maintained for 8 min; finally, in the third step, the temperature increased 10 °C/min until 300 °C and kept constant for 2 min. The identification of the different compounds was performed through the Library of the National Institute of Standards (NIST).

#### 3.3.3. Determination of the Total Phenolic Content (TPC)

The total phenolic content of the *Curcuma longa* L. oil was measured according to the method described by Fernández-Marín et al. [35] with slight modifications. Briefly, 0.025 g of each extract was dissolved in 3 mL of methanol and centrifuged at 450 rpm for 24 h. The supernatant was then collected and a 300 μL solution of each extract was used. The measurement was carried out at 760 nm using an UV/Vis spectrophotometer (V-630, Jasco, Pfungstadt, Germany). Gallic acid was used as the reference standard. The results were expressed as mg gallic acid equivalents/g of extracted *Curcuma longa* L. oil (mg GAE/g). Each sample was analysed in triplicate.

#### 3.3.4. Antioxidant Activity

The antioxidant activity of the extracted *Curcuma longa* L. oil was evaluated using three different assays: 2,2-diphenyl-1-picrylhydrazyl (DPPH) radical scavenging assay; ferric ion reducing antioxidant power (FRAP) assay; and 2,2′-azinobis(3-ethylbenzothiazoline-6-sulfonic acid) (ABTS) radical scavenging assay. The samples were prepared by dissolving 0.025 g of *Curcuma longa* L. oil in 3 mL of methanol (MeOH) and centrifuged at 450 rpm for 24 h. The supernatant was then collected for use in the antioxidant assays.

##### DPPH Radical Scavenging Assay

The antioxidant activity of *Curcuma longa* L. oil was evaluated by the DPPH (2,2-diphenyl-1-picrylhydrazyl) free radical elimination assay according to the method described by Fernandez-Marin et al. [35] with slight modifications. Three hundred μL of supernatant (prepared as described before) from each sample was mixed with 3 mL of a DPPH solution (6 × 10^−5^ M) in MeOH. After 15 min, the decrease in absorbance was measured at 515 nm with UV/Vis spectrophotometer (Jasco V-630, Pfungstadt, Germany). Trolox was used as standard and the results were expressed in mg Trolox equivalent/g of *Curcuma longa* L. oil (mg TE/g). Each sample was measured in triplicate.

##### FRAP Assay

The FRAP assay, consists of measuring the reduction of ferric-tripyridyltriazine (Fe^III^–TPTZ) to a ferrous complex (ferrous-tripyridyltriazine, Fe^II^–TPTZ) by the presence of an antioxidant compound with an acetate buffer at pH 3.6. In the process a colour variation (blue-colored product) occurs which is measured with a UV/Vis spectrophotometer at 593 nm (Jasco V-630). From each sample, 300 μL of the supernatant (prepared as described before) were used and mixed with 3 mL of the FRAP reagent solution. This last solution was prepared by using 2.5 mL of 2,4,6-tripyridyl-s-triazine (10 mM), 25 mL of acetate buffer (300 mM) at pH 3.6, in HCl (37% *v*/*v*) and 2.5 mL of FeCl_3_-6H_2_O (20 mM) in distilled water [36]. Trolox was applied as reference and the results were expressed in mg Trolox equivalent/g of *Curcuma longa* L. oil extracted (mg TE/g). Each sample was tested in triplicate.

##### 2,2′-Azino-bis(3-ethylbenzothiazoline-6-sulfonic acid) Assay

The ABTS assay is based on the reaction of a solution of ABTS (7 mM) with potassium persulphate (2.45 mM) in the dark to generate the ABTS+ radical. This solution was diluted by a phosphate buffer salt (PBS) solution at pH 7.4 until an absorbance of 0.7 at 734 nm. Then, 30 µL of supernatant of each sample were added to a 3 mL of the last solution. After 6 min, the absorbance was measured by a UV/Vis spectrophotometer (Jasco V-630) [36]. Trolox was the standard reference and results were presented in mg Trolox equivalent/g *Curcuma longa* L. oil extracted (mg TE/g). All samples were measured in triplicate.

### 3.4. Statistical Analysis

The statistical analysis was performed using IBM SPSS software (Version 24, Inc. Chicago, IL, USA). One-way analysis of variance (ANOVA) was used to validate the experimental design and to study the differences between the MAE and Soxhlet extraction for each analysed parameter. The values of the significant differences were identified by Duncan’s multiple range test and significance was accepted at α = 0.05. The results were shown as mean ± SD (standard deviation) by performing 3 measurements for each analysis.

## 4. Conclusions

The optimization of the extraction yield of *Curcuma longa L.* oil was carried out by microwave-assisted extraction using the Box-Behnken experimental design with response surface methodology. The optimum conditions for the extraction were 29.99 min, 160 W and a *Curcuma longa* L./EtOH ratio of 1:20 g/mL, and the yield of extracted oil was 10.32 ± 0.69%. This yield value was higher than that obtained by the conventional Soxhlet method (8.44 ± 0.17%).

It was also concluded that the treatments applied for the extraction of the *Curcuma longa* L. oil influenced the TPC and antioxidant activity. The data from the three applied assays (DPPH, ABTS and FRAP) revealed higher phenolic content and higher antioxidant activity (in *Curcuma longa* L. oil when it was extracted using the MAE method.

Therefore, these results demonstrated that MAE is a viable and more environmentally friendly method for the extraction of *Curcuma longa* L. oil.

## Figures and Tables

**Table 1:** Box-Behnken experimental design and operational conditions represented in accordance of dimensional and dimensionless independent variables t (time, min) (X_1_); P (power, W) (X_2_), R (ratio *w*/*v*, g/mL) (X_3_) and experimental response (extraction yield by microwave-assisted extraction, Y_C-MAE_).

Experiments	Independent Variables			Normalized Variables			Extraction Yield
	t (min)	P (W)	R (g/mL)	X_1_	X_2_	X_3_	Y_C-MAE_ %
**1**	20	200	1:8	0	0	0	9.50
**2**	30	150	1:8	1	−1	0	8.59
**3**	30	200	1:5	1	0	1	4.00
**4**	10	150	1:8	−1	−1	0	5.58
**5**	30	250	1:8	1	1	0	6.92
**6**	10	200	1:5	−1	0	1	7.06
**7**	10	200	1:20	−1	0	−1	7.41
**8**	20	250	1:20	0	1	−1	4.48
**9**	20	200	1:8	0	0	0	9.35
**10**	10	250	1:8	−1	1	0	6.12
**11**	20	150	1:20	0	−1	−1	10.84
**12**	20	250	1:5	0	1	1	3.03
**13**	30	200	1:20	1	0	−1	9.27
**14**	20	200	1:8	0	0	0	9.47
**15**	20	150	1:5	0	−1	1	6.77

**Table 2:** Regression coefficients and statistical parameters of the optimization of *Curcuma longa L.* oil extraction by MAE.

Coefficients	Y_C-MAE_
**b_0_**	9.44
**b_1_**	0.32
**b_2_**	−1.40 ^a^
**b_3_**	−1.39 ^a^
**b_12_**	−0.55
**b_13_**	−1.23
**b_23_**	0.66
**b_11_**	−0.99
**b_22_**	−1.65 ^b^
**b_33_**	−1.51
**R^2^**	0.82
**F-exp**	2.55
**F-critical**	0.16
**Significance level (%)**	84.24

F-critical for nine degrees of freedom. ^a^ Significant coefficients at the 95% confidence level. ^b^ Significant coefficients at the 90% confidence level.

**Table 3:** The dimensionless and dimensional optimum points and the predicted and experimental extraction yield by microwave-assisted extraction (Y_C-MAE_) and the extraction yield by the Soxhlet method (Y_C-S_). The experimental extraction yields were mean ± standard deviation of three replications (n = 3).

MAE					Soxhlet
**X_1_ (t, min)**	**X_2_ (P, W)**	**X_3_** **(R, g/mL)**	**Y %** **(Predict Value)**	**Y_C-MAE_ % (Experimental Value)**	**Y_C-S_ %** **(Experimental Value)**
**0.99 (29.99)**	−0.79 (160.41)	−0.99 (1:20)	10.92	10.32 ± 0.69	8.44 ± 0.17

**Table 4:** *Curcuma longa* L. oil composition evaluated by GC/MS.

No.	Component	MAE		Soxhlet	
		A (%)	RT (min)	A (%)	RT (min)
**1**	α-curcumene	0.55	18.8991	0.92	18.8993
**2**	Zingiberene	0.35	19.1652	0.54	19.1654
**3**	β-Sesquiphellandrene	0.65	19.7682	0.95	19.7684
**4**	ar-Tumerol	0.74	20.859	0.84	20.8592
**5**	*p*-Cymene	1.52	21.329	1.48	21.3293
**6**	Zingiberenol	0.26	21.7725	0.32	21.6929
**7**	3-Ethyl-N-methylaniline	0.51	21.8434	0.44	21.8436
**8**	ar-Turmerone	33.78	22.3933	37.08	22.3935
**9**	Tumerone	20.12	22.4553	18.15	22.4555
**10**	Bisacurol	0.57	22.7657	0.25	22.7482
**11**	β-Tumerone	20.05	22.9786	19.22	22.9788
**12**	(Z)-α-Atlatone	0.52	23.1471	0.50	23.1473
**13**	(6*R*, 7*R*)-bisabolone	0.96	23.6437	1.00	23.6439
**14**	(*E*)-α-Atlatone	3.01	24.0516	2.38	24.0519
**15**	(o)-Paradol	0.66	24.6902	0.94	25.6861
**16**	2-Cyclohenen-1-one,6-[(1*S*)-1,5-dimethyl-3-oxo-4-hexen-1-yl]-3-methyl-(6*S*)	0.68	25.8697	0.67	25.8699

A: Area; RT: retention time; MAE: Microwave-Assisted Extraction S: Soxhlet.

**Table 5:** Results of total phenolic content (TPC) and antioxidant activity (DPPH, FRAP and ABTS assays) of the *Curcuma longa* L. oil extraction by MAE (microwave-assisted extraction technique) and Soxhlet. Values were mean ± standard deviation (n = 3). Superscript letters represent significant differences (Duncan’s test, *p* < 0.05) between each assay with the two extraction methods.

	MAE	Soxhlet
**TPC (mg GAE/g)**	232.75 ± 0.31 ^a^	140.72 ± 0.42 ^b^
**DPPH (mg TE/g)**	64.71 ± 0.49^a^	49.00 ± 0.33 ^b^
**FRAP (mg TE/g)**	255.66 ± 0.24 ^a^	73.37 ± 0.18 ^b^
**ABTS (mg TE/g)**	79.82 ± 0.03 ^b^	71.42 ± 0.04 ^a^

GAE: gallic acid equivalents; TE: Trolox equivalent.

**Table 6:** List of the experimental variables involved in the microwave-assisted extraction of *Curcuma longa* L. oil.

Variables		Nomenclature	Units	Value or Range
**Independents**	Reaction Time Microwave Power Ratio *w*/*v* (*Curcuma longa* L./EtOH)	t P R	min W g/mL	10–30 150–250 1:20–1:5
**Dependent**	Curcuma oil yield	Y	%	−

## Data Availability

Data is contained within the article.

## Sample Availability

Samples of the compounds are not available from the authors.
